# Performance of 3 large language models in detecting urinary formed elements

**DOI:** 10.1097/MD.0000000000049041

**Published:** 2026-06-05

**Authors:** Xiaoning Zeng, Sirou Li

**Affiliations:** a Department of Clinical Laboratory Medicine, National Cancer Center/National Clinical Research Center for Cancer/Cancer Hospital & Shenzhen Hospital, Chinese Academy of Medical Sciences and Peking Union Medical College, Shenzhen, China.

**Keywords:** Chat-GPT, Deepseek, Gemini, large language model, urine formed elements

## Abstract

The integration of large language models (LLMs) into medical laboratories has gained significant attention, especially in the field of image recognition. However, research on their ability to identify unstained images, such as those of urinary formed elements, remains scarce. This study assesses the image recognition capabilities of 3 LLMs, ChatGPT-4o, Deepseek Janus Pro7B and Google Gemini, in detecting urinary formed elements. This cross-sectional study analyzed 45 urine morphology images, utilizing a standardized prompt to guide ChatGPT-4o, Deepseek Janus Pro7B and Google Gemini in recognizing urine formed elements. Each image was independently evaluated 3 times, and the results were assessed using a 5-point Likert scale. The accuracy and consistency of the models were compared through the Friedman test, Kendall W score, and the Mann–Whitney U test. Gemini Advanced demonstrated superior performance with a 31% accuracy rate. ChatGPT-4o (W = 0.797) and Gemini (W = 0.812) exhibited strong consistency, suggesting a superior performance compared to Deepseek (W = 0.663). Statistical analysis revealed a significant difference in performance between the 3 models, with Gemini showing superior ability to identify urinary formed elements, particularly cast and microorganism. While ChatGPT-4o, Deepseek Janus Pro7B and Google Gemini show promise and exhibit potential in identifying urinary formed elements, their diagnostic performance remains limited and are currently inadequate for clinical use in identifying urine morphology. To enhance their clinical applicability, further improvements in training data and model optimization are required. Future research should focus on enhancing these models’ performance to ensure their broader utility in medical laboratories.

## 1. Introduction

The development of artificial intelligence (AI) has consistently captured global attention. In 2022, OpenAI introduced Chat-GPT, a large language model (LLM) based on the Transformer architecture, which marked a significant advancement in deep learning for language understanding and generation. The latest iteration in the Chat-GPT series, GPT-4o, further improves its reasoning capabilities and multimodal processing abilities. Although LLMs offer considerable advantages, training these models demands substantial financial and computational resources. Google Gemini AI represents a major technological breakthrough, showcasing advanced functionalities and innovative features. Gemini incorporates multimodal capabilities, enabling it to process text, audio, and video, making it especially valuable in medical imaging.^[1]^ In 2025, the Chinese company DeepSeek released its first language reasoning model, Deepseek-R1. In contrast to previous models, Deepseek significantly reduced both the training costs and time, demonstrating its efficient resource utilization. Furthermore, the R1 model is trained using reinforcement learning, enhancing its mathematical and logical reasoning capabilities.

With the development of LLM, an increasing number of clinical practitioners are utilizing these tools to assist in clinical decision-making.^[2]^ Research has demonstrated that ChatGPT holds considerable value in the diagnosis and treatment of cancer.^[3,4]^ However, in terms of drug-related issues, ChatGPT-4o faces challenges regarding accuracy and completeness.^[5]^ Additionally, LLMs are increasingly being used in medical education, such as in standardized resident training and problem-based learning.^[6,7]^ Presently, ChatGPT is widely applied across various domains of clinical medicine, including the development of rehabilitation programs, injury assessments, preoperative planning for joint replacements, and more.^[8,9]^ Different models show considerable variation in their clinical applications. In breast cancer clinical decision-making, both GPT-4 and Claude2 excel in diagnostic assessments, with GPT-4 being the most effective in terms of response relevance and clinical applicability, while GPT-3.5 lags behind in all areas.^[3]^ Gemini technological prowess lies in its capacity to analyze intricate datasets, including charts and images. In a research endeavor within the ophthalmology domain, Gemini exhibited commendable performance.^[10]^ In the task of identifying urological conditions from cystoscopic images, ChatGPT demonstrated a slight performance advantage over Claude.^[11]^ Deepseek surpasses GPT-4 in diagnosing complex oral cases.^[12]^ Although Deepseek exploration in the medical field is currently limited due to its later release, its open-source and reasoning-based approach offers considerable potential.^[9]^

The use of LLMs in clinical laboratories is increasingly prevalent, particularly in the field of image recognition. Several studies have demonstrated ChatGPT proficiency in recognizing blood cell morphology, achieving 100% accuracy in identifying Howell-Jolly bodies and Auer rods.^[13,14]^ It is worth noting that no studies have yet assessed the ability of large language models to recognize unstained images. To address this gap, we assessed the capabilities of 3 different LLMs in recognizing urinary formed elements. This study aims to explore the image recognition capabilities of LLMs and compare the performance of these 2 models, thereby broadening the potential applications of large language models in medical laboratories.

## 2. Method

### 2.1. Study design

This study presents a cross-sectional analysis in which we examine 45 urine morphology images to evaluate the performance of ChatGPT-4o, Deepseek Janus Pro7B and Google Gemini in identifying urine formed elements. A standardized prompt was used to guide 3 models in analyzing the images to ensure their outputs accurately met the requirements of the image recognition task. The command provided to the models was as follows: “You are an expert in urine morphology with in-depth knowledge of the morphological features of urine formed elements used in diagnosis. I will now send you images for recognition and description. Please identify and describe the urine formed elements indicated in the images. Are you ready? This is a microscopic image of a urine sediment; please identify the object pointed to by the arrow.” To minimize the impact of individual differences, 2 experts independently evaluated the models’ outputs. Recognizing that large language models may generate varied responses based on minor input differences, we performed 3 independent queries for each image and assessed and scored each output. The entire results are shown in the Table S1.

We evaluated the results using a 5-point Likert scale based on the following criteria: 1 point indicates complete inaccuracy, such as identifying cells as crystals when this is unrelated to the correct answer. 2 points denote a fundamental error with only partial accuracy in the description, for example, correctly identifying microorganisms but confusing bacteria with fungi, or identifying only red blood cells in a red blood cell cast. 3 points indicate that the major classification is correct, but finer details are inaccurate, such as mistaking calcium oxalate crystals for uric acid crystals or failing to describe certain features, such as identifying clue cells as epithelial cells. 4 points suggest that most of the description is accurate, but there are deviations in content, such as confusing red blood cell casts with blood casts. 5 points indicate complete and accurate identification.

### 2.2. Data resources

The image data used in this study were sourced from the External Quality Assessment (EQA) program for urine morphology in China, conducted from 2021 to 2023. These images were provided by the Clinical Laboratory Center of the National Health Commission of China and encompass a range of typical and abnormal morphologies of formed elements in urine (Table 1). The images were acquired using standardized procedures for urine sample collection, processing, and microscopic examination, ensuring the reliability and consistency of the image quality, which accurately represents the morphological characteristics of the formed elements in urine.

**Table 1:** Pairwise comparisons of chatbots’ scores.

Chatbot	Deepseek	Chatgpt-4o	Gemini
Deepseek	—	1.14 × 10^−9^	6.23 × 10^−17^
Chatgpt-4o	1.14 × 10^−9^	—	8.70 × 10^−5^
Gemini	6.23 × 10^−17^	8.70 × 10^−5^	—

The EQA project aims to evaluate the accuracy and consistency of clinical laboratories across the country in conducting urinary morphological testing. Participating laboratories identify and report the types and quantities of formed elements in urine based on the provided images, following established standards and procedures. These standards include methods for classifying and counting urinary cells, casts, and other components, as well as guidelines for identifying and reporting abnormal elements. Image evaluations are performed by experienced clinical laboratory experts, whose results serve as the gold standard for model assessment in this study.

### 2.3. Study analysis

We used R (version 4.3.3) to analyze the identification results of ChatGPT-4o, Deepseek Janus Pro7B and Google Gemini. In this study, the Friedman test and Kendall W score were used to assess the consistency of results within the same model across 3 trials, while the Mann–Whitney U test was applied to determine whether there were significant differences in accuracy and scoring among 3 models. Finally, we classified the urinary formed elements into 6 categories: cells, crystals, casts, microorganisms, parasites and others. A classification analysis was performed on the models’ performance in recognizing different types of urinary formed elements, to understand the strengths and weaknesses of different models in identifying various components. A *P*-value of < .05 was considered statistically significant.

## 3. Result

**Figure 1.**
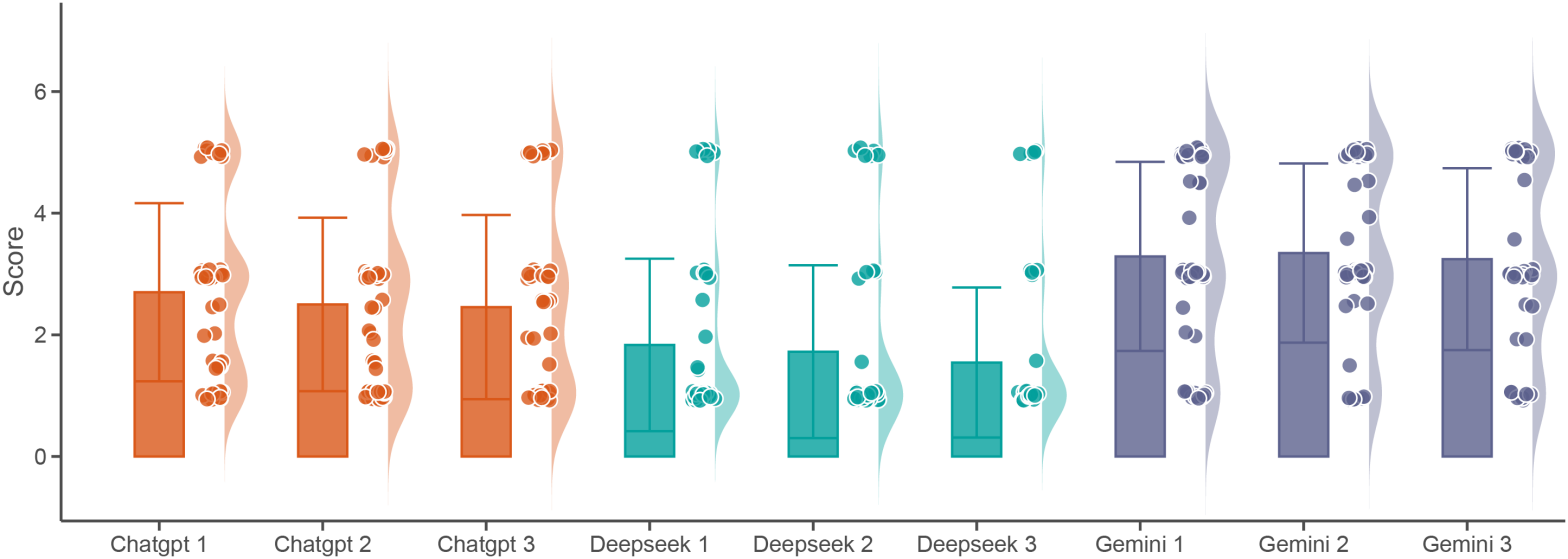
Distribution cloud chart of the scores for 3 LLMs. The x-axis represents the models and the number of queries, and the y-axis represents the scores.

**Figure 2.**
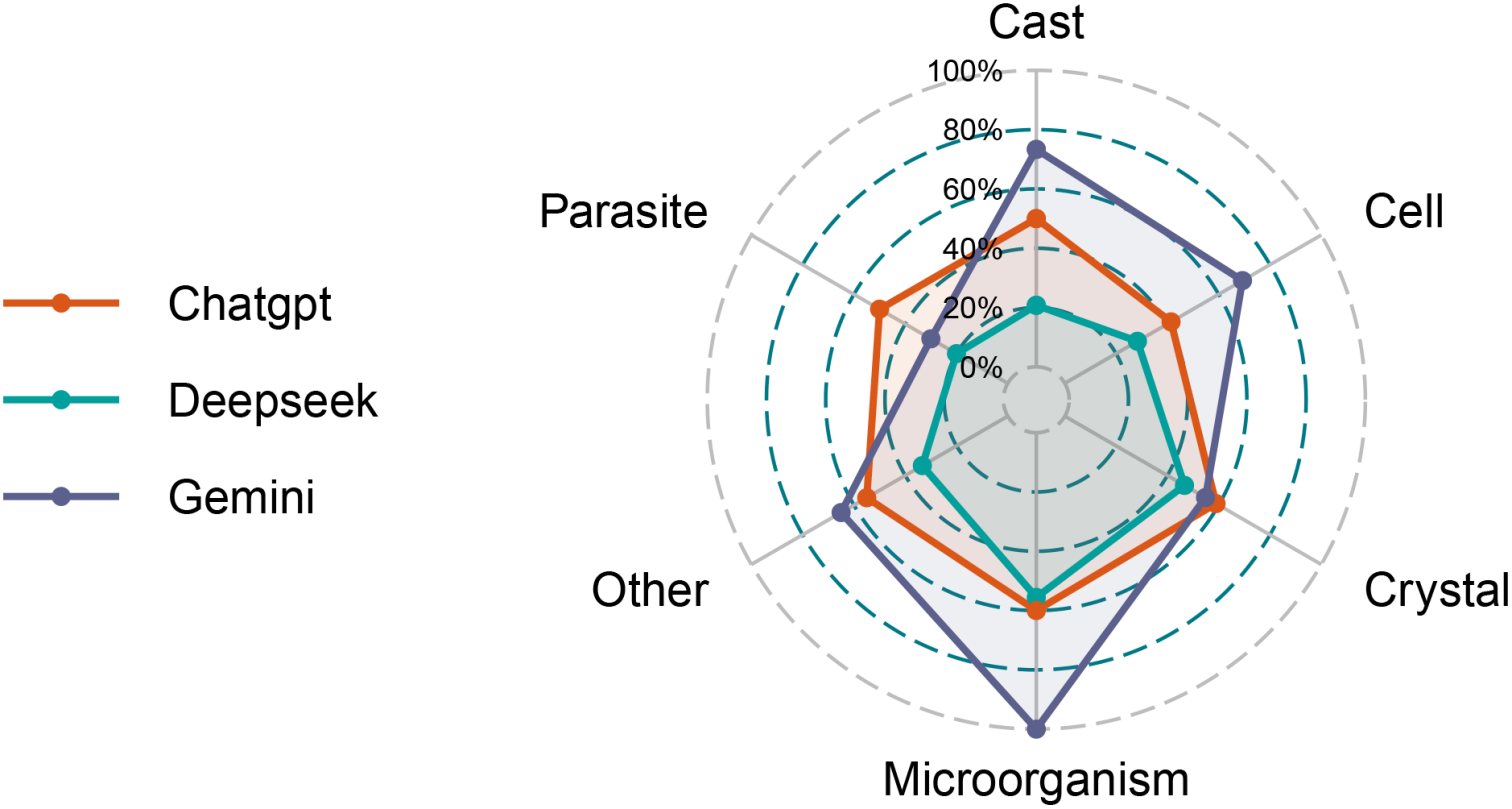
Radar chart of the scores rate for 3 LLMs.

Table 1 shows the scores and score rates of ChatGPT-4o, Deepseek and Gemini in different categories of images. Among 3 LLMs, Gemini won the highest accuracy (31%), follewed by ChatGPT-4o (29%), while Deepseek got the lowest correct rate (13%). The results were rated using the Likert scale, as shown in Figure 1. Friedman test revealed no significant differences between the 3 evaluations of 3 LLMs, with p-values of 0.089, 0.099 and 0.812, respectively. The consistency of ChatGPT-4o(W = 0.797) and Gemini(W = 0.812) was strong, indicating a better performance than Deepseek(W = 0.663). After applying the Mann–Whitney U test, we found a significant difference among these 3 models’ ability to identify urinary formed elements, as shown in Table 1, indicating that Gemini has a distinct advantage in practical applications. Figure 2 shows the scores of 3 models for different components. Overall, Gemini achieves higher scores, especially in microorganism, while ChatGPT-4o have an advantages in parasite and crystal. The specific score details are presented in Table 2.

**Table 2:** Scores of 3 AI chatbots in different categories of images.

EQA	Type	Number	Deepseek Score	Chatgpt Score	Gemini Score
Red blood cell casts	Cast	1	3	9	9
Red blood cell casts or mixed cell casts	Cast	1	4	3	10
Granular tube type	Cast	3	9	30	36
Wax sample tube type, wide tube type	Cast	1	3	5	9
Transparent tube type	Cast	3	9	20.5	35
White blood cell	Cell	3	21.5	16.5	31
Red blood cell	Cell	3	10.5	21.5	36.5
Squamous epithelial cells	Cell	2	7.5	17.5	30
Renal tubular epithelial cells	Cell	1	3	3	5.5
Phagocyte	Cell	1	3	3	3
Transitional epithelial cells (lower layer)	Cell	1	5	8.5	10.5
Transitional epithelial cells (middle layer)	Cell	3	9	17	29
Calcium oxalate crystals	Crystal	3	21	27	21
Cholesterol crystals	Crystal	1	5	9	9
Magnesium ammonium phosphate crystals (triphosphate)	Crystal	2	12	22	20
Calcium phosphate crystallization	Crystal	2	6	12	12
Equuric acid crystals	Crystal	1	3	7	9
Uric acid crystals	Crystal	3	39	35	27
Sodium urate crystals	Crystal	1	5	3	9
Bacteria	Microorganism	1	7	15	15
Fungus	Microorganism	2	18	12	30
Sperm	Other	1	11	15	15
Clue cell	Other	2	6	14.5	9
Mucus thread	Other	1	3	3.5	15
Protozoa	Parasite	2	6	15	9

Clinical Laboratory Center of the National Health Commission of China.

Total images: n = 45.

## 4. Discussion

This study investigates the application of LLMs in medical laboratories, focusing specifically on the performance of 3 models, ChatGPT-4o, Deepseek and Gemini, in recognizing urinary formed elements in images. Numerous studies have explored the use of ChatGPT in medical laboratory education, including applications in histopathological descriptions,^[15]^ abnormal blood cell morphology,^[13,14]^ thyroid nodule ultrasound images,^[16]^ and radiation images.^[17]^ Research shows that Gemini exhibited superior performance in comprising images sourced from the Israel Ophthalmology Resident Examination.^[10]^ However, research on the application of Deepseek in medical laboratory education, particularly in medical image analysis, remains limited. Given the lack of specific studies on the performance of ChatGPT-4o, Deepseek and Gemini in identifying urinary formed elements, evaluating their capabilities in this domain is crucial. This research aims to assess the potential of AI-assisted methods for helping patients and healthcare professionals identify and interpret medical reports more effectively.

This study demonstrates that Gemini attains a 100% accuracy rate in identifying Squamous epithelial cells, Bacteria, Fungus, Sperm, and Mucus threads. ChatGPT-4o accurately recognizes bacteria and sperm, achieving 100% accuracy in these images. The recognition performance of Deepseek Janus Pro 7B for bacteria and sperm is also satisfactory. In addition, ChatGPT-4o is superior to the other 2 in identifying Crystal and parasite. However, the accuracy of the 3 models in identifying images is still needed to improve. For example, ChatGPT-4o misidentified red blood cells as hyaline casts (Table 3), suggesting potential issues with contour identification. Deepseek Janus Pro 7B incorrectly identified red blood cells as White blood cells (Table 3), despite the absence of interfering particles in their cytoplasm, indicating a misleading error in identification. Equally confusing is Gemini identification of red blood cells as renal tubular epithelial cells, which was due to a mistaken recognition of the presence of a nucleus within the cell. Furthermore, ChatGPT-4o confused granular casts with hyaline casts (Table 3), while Deepseek Janus Pro 7B mistakenly identified granular casts as bacteria (Table 3), and Gemini incorrectly classified granular casts as waxy casts. These errors demonstrate incorrect identification of the cast contents. In terms of parasite identification, ChatGPT-4o misclassified protozoa as renal tubular epithelial cells (Table 3), while Deepseek Janus Pro 7B identified protozoa as uric acid crystals (Table 3), and Gemini incorrectly classified protozoa as White blood cells. Given the lack of specific clinical context and the static nature of the images, confusion with other visible urine components is understandable. ChatGPT-4o, Deepseek Janus Pro 7B and Gemini face significant challenges in accurately identifying parasites.

**Table 3:**
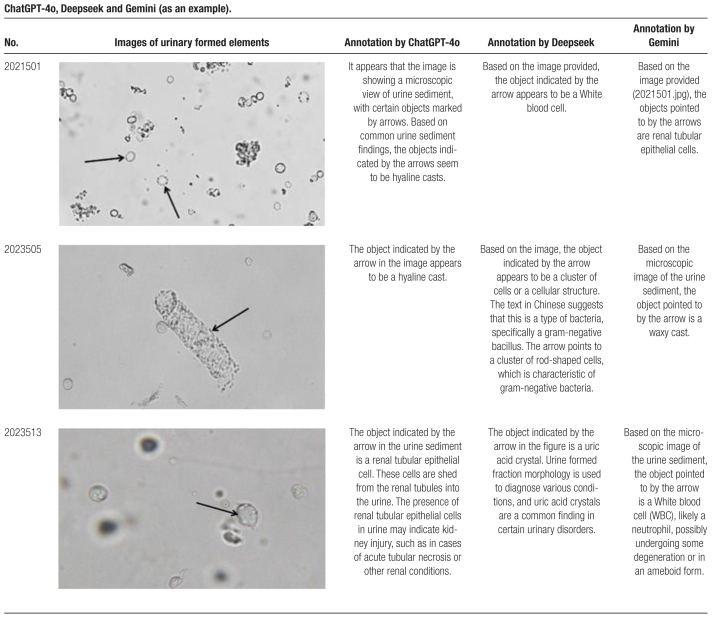
ChatGPT-4o, Deepseek and Gemini (as an example).

Unfortunately, ChatGPT-4o, Deepseek Janus Pro 7B, and Gemini exhibit relatively low overall accuracy in identifying urine-forming elements. However, Gemini demonstrates better recognition performance among them. The results suggest that ChatGPT-4o, Deepseek Janus Pro 7B, and Gemini cannot replace human expertise in identifying urine morphology, highlighting the urgent need to further enhance the models’ recognition capabilities.

However, this study has several limitations. First of all, the questions provided by the National Clinical Laboratory Center of China mainly involve the direct query of ChatGPT-4o, Deepseek Janus Pro 7B and Gemini based on urine tangible component images, with no specific cases or additional clinical information provided. As a result, the accuracy of ChatGPT-4o, Deepseek Janus Pro 7B and Gemini remains uncertain due to the lack of sufficient clinical background. Furthermore, this preliminary study only focused on ChatGPT-4o, Deepseek Janus Pro 7B and Gemini as experts in urine tangible component morphology, without utilizing a broader range of cell morphology data sets for training. Additionally, the overall sample size is limited to 45 cases, which is relatively small. To more comprehensively evaluate the performance of ChatGPT-4o, Deepseek Janus Pro 7B and Gemini, a larger sample size is needed.

In summary, although ChatGPT-4o, Deepseek and Gemini show considerable potential, they are not reliable tools for morphological identification of urinary formed elements. Our research establishes a baseline for future advancements in multimodal LLM and highlights the importance of continuous development to ensure reliability in medical laboratory applications.

## 5. Conclusion

This study demonstrates that LLMs, especially ChatGPT-4o, Deepseek and Gemini, have the potential to recognize the morphology of urinary formed elements. However, their diagnostic performance is currently limited. This highlights the inherent limitations of these models in medical laboratory image recognition tasks, and highlights the cautious attitude of medical professionals towards their application in clinical environments. In order to effectively integrate LLM into the medical laboratory diagnostic workflow, future work should focus on optimizing the LLM architecture of medical imaging, expanding the training data set, and improving its diagnostic ability and reliability in clinical practice.

## Acknowledgments

We wish to express our gratitude to the Clinical Laboratory Center of the National Health Commission of China for their provision of the images and kind permission to utilize them.

## Author contributions

**Conceptualization:** Xiaoning Zeng, Sirou Li.

**Formal analysis:** Xiaoning Zeng.

**Writing – original draft:** Xiaoning Zeng, Sirou Li.

**Writing – review & editing:** Xiaoning Zeng, Sirou Li

**Data curation:** Sirou Li.


